# Intestinal persistence of *Bifidobacterium infantis* is determined by interaction of host genetics and antibiotic exposure

**DOI:** 10.1093/ismejo/wrae107

**Published:** 2024-06-19

**Authors:** Yiming Wang, Jocelyn M Choo, Alyson C Richard, Lito E Papanicolas, Steve L Wesselingh, Steven L Taylor, Geraint B Rogers

**Affiliations:** Microbiome and Host Health Programme, South Australian Health and Medical Research Institute, Adelaide, South Australia 5001, Australia; Infection and Immunity, Flinders Health and Medical Research Institute, College of Medicine and Public Health, Flinders University, Bedford Park, South Australia 5042, Australia; Microbiome and Host Health Programme, South Australian Health and Medical Research Institute, Adelaide, South Australia 5001, Australia; Infection and Immunity, Flinders Health and Medical Research Institute, College of Medicine and Public Health, Flinders University, Bedford Park, South Australia 5042, Australia; Microbiome and Host Health Programme, South Australian Health and Medical Research Institute, Adelaide, South Australia 5001, Australia; Infection and Immunity, Flinders Health and Medical Research Institute, College of Medicine and Public Health, Flinders University, Bedford Park, South Australia 5042, Australia; Microbiome and Host Health Programme, South Australian Health and Medical Research Institute, Adelaide, South Australia 5001, Australia; Infection and Immunity, Flinders Health and Medical Research Institute, College of Medicine and Public Health, Flinders University, Bedford Park, South Australia 5042, Australia; SA Pathology, SA Health, Adelaide, South Australia 5001, Australia; Microbiome and Host Health Programme, South Australian Health and Medical Research Institute, Adelaide, South Australia 5001, Australia; Microbiome and Host Health Programme, South Australian Health and Medical Research Institute, Adelaide, South Australia 5001, Australia; Infection and Immunity, Flinders Health and Medical Research Institute, College of Medicine and Public Health, Flinders University, Bedford Park, South Australia 5042, Australia; Microbiome and Host Health Programme, South Australian Health and Medical Research Institute, Adelaide, South Australia 5001, Australia; Infection and Immunity, Flinders Health and Medical Research Institute, College of Medicine and Public Health, Flinders University, Bedford Park, South Australia 5042, Australia

**Keywords:** microbiome, secretor status, α(1, 2)-fucosylated glycans, *Bifidobacterium*, *Bifidobacterium infantis*

## Abstract

Probiotics have gained significant attention as a potential strategy to improve health by modulating host–microbe interactions, particularly in situations where the normal microbiota has been disrupted. However, evidence regarding their efficacy has been inconsistent, with considerable interindividual variability in response. We aimed to explore whether a common genetic variant that affects the production of mucosal α(1,2)-fucosylated glycans, present in around 20% of the population, could explain the observed interpersonal differences in the persistence of commonly used probiotics. Using a mouse model with varying α(1,2)-fucosylated glycans secretion (*Fut2*^WT^ or *Fut2*^KO^), we examined the abundance and persistence of *Bifidobacterium* strains (*infantis, breve,* and *bifidum*). We observed significant differences in baseline gut microbiota characteristics between *Fut2*^WT^ and *Fut2*^KO^ littermates, with *Fut2*^WT^ mice exhibiting enrichment of species able to utilize α(1,2)-fucosylated glycans*.* Following antibiotic exposure, only *Fut2*^WT^ animals showed persistent engraftment of *Bifidobacterium infantis*, a strain able to internalize α(1,2)-fucosylated glycans, whereas *B. breve* and *B. bifidum*, which cannot internalize α(1,2)-fucosylated glycans, did not exhibit this difference. In mice with an intact commensal microbiota, the relationship between secretor status and *B. infantis* persistence was reversed, with *Fut2*^KO^ animals showing greater persistence compared to *Fut2*^WT^. Our findings suggest that the interplay between a common genetic variation and antibiotic exposure plays a crucial role in determining the dynamics of *B. infantis* in the recipient gut, which could potentially contribute to the observed variation in response to this commonly used probiotic species.

## Introduction

Host–microbiome interactions play a pivotal role in shaping human physiology. The intestinal microbiome in particular is an important regulator of innate and adaptive immunity [1], metabolic control [2], the central nervous system [3], as well as contributing to energy and nutrient harvest [4], and suppressing pathogen proliferation [5]. Given the association between disruption of the commensal gut microbiota and adverse outcomes, there is significant interest in approaches that facilitate its restoration following perturbation. Among the most well-established of these approaches is the ingestion of viable commensal bacteria in the form of probiotics.

Probiotics can be defined as “live microorganisms, which when administered in adequate amounts, confer a health benefit on the host” [6]. Most commonly, these take the form of individual strains or multistrain consortia, of well-characterized commensal bacteria, prepared either as liquid suspensions or in freeze-dried capsules. The principal concept underlying the use of probiotics is that the introduction of live bacteria can re-establish physiological homeostasis by modifying the composition or behaviour of the gut microbiota or by directly providing regulatory cues to the host. Despite substantial evidence supporting the efficacy of probiotics in principle, their use remains poorly supported by empirical data in many physiological or health contexts [7, 8]. Further, substantial interindividual variance in probiotic persistence has been noted, in part explained by variation in colonization resistance by the microbiome [9, 10]. Consequently, the global probiotics market, which is projected to reach USD 73.9 billion by 2030 [11], is dominated by direct-to-consumer sales, with little or no consideration is given to recipient traits that might substantially influence probiotic efficacy.

Various mechanisms enable the human gut to regulate commensal microbiota composition. One of the principal mechanisms involves the secretion of specific types of sugars that are utilized by beneficial microbial species. Many mucosal constituents and secreted factors are decorated with glycans (oligosaccharides), which are added by a diverse family of glycosyltransferase enzymes [12]. Of these, the *FUT2* gene encodes a galactoside α(1,2)-fucosyltransferase, which adds an L-fucose monosaccharide to nonreducing end Gal residues to form Fucα1-2Gal-O-R glycans, termed the H antigens [13, 14]. Expressed by multiple mucosal epithelial cell types, this H antigen is a highly versatile structure that can be further modified to form many other important glycans, including the AB blood group glycans. Because *FUT2* controls the nature of the various α(1,2)-fucosylated glycans secreted by mucosal surfaces, it is commonly referred to as the “secretor” gene [13].

Across the human population, multiple nonsense single-nucleotide polymorphisms (SNPs) are found within the *FUT2* gene [15], leading to a “nonsecretor” phenotype. The nonsecretor phenotype, like the AB blood groups, is one of the more common functional mutations maintained in the population, with approximately one-fifth of people carrying homozygous loss-of-function *FUT2* genes [15]. This high carriage of loss-of-function mutations is likely a result of positive selection from altered susceptibility to infections by certain bacterial and viral pathogens [16]. However, as fucosylated glycans are an important nutrient source for gut microbes, their absence in nonsecretors has been shown to influence the composition of commensal microorganisms [16, 17].

The intact commensal microbiota of secretor individuals is likely to be enriched for glycan-utilizing bacteria. In contrast, depletion of commensal taxa, for example, through antibiotic exposure, can provide a selective advantage to exogenous glycan utilizers that are absent in nonsecretors [9, 18]. Probiotic preparations typically contain *Bifidobacteria* (*Bifidobacterium adolescentis*, *animalis*, *bifidum*, *breve,* and *longum*) and/or Lactobacilli (*Lactobacillus acidophilus*, *casei*, *fermentum*, *gasseri*, *johnsonii*, *paracasei*, *plantarum*, *rhamnosus,* and *salivarius*). Both genera include species that encode the specific glycoside hydrolases (GHs), GH29, GH95, and GH151, which can utilize the H antigen. However, both genera also include species without this glycoside hydrolase capacity. Therefore, the ability of a probiotic to colonize and persist in an individual may depend on the presence of secreted glycans and the ability of the introduced bacterial strain to utilize them. This is supported by studies identifying increased persistence of such glycan-utilizing species when supplemented with exogenous oligosaccharides [19, 20].

We hypothesized that the interplay between secretor status, the glycan utilization ability of the probiotic strain, and the presence of a disrupted commensal microbiota due to antibiotic exposure would collectively influence the abundance and persistence of probiotic populations in the gut. To test this hypothesis, we introduced probiotic *Bifidobacterium* strains into a murine model of secretor/nonsecretor status, with or without prior antibiotic depletion of commensal microbiota.

## Materials and methods

Details of reagent catalogue numbers and resource links are provided in Supplementary Table S1.

### Mouse model

#### Establishment of a Fut2 knockout mice

A *Fut2*^KO^ mouse line was developed using CRISPR/Cas9 technology in C57BL/6 mice (IMSR_JAX:000664) by South Australian Genome Editing (SAGE). Briefly, a 1230-bp region of the *Fut2* exon region was excised using targeted CRISPR guide sequences. Gene knock out was confirmed by Sanger sequencing and phenotype confirmed by α(1,2)-fucosylated glycan staining of intestinal biopsies using *Ulex Europaeus* lectin 1 (Supplementary Fig. S1), as described previously [21]. Littermate *Fut2*^WT^ and *Fut2*^KO^ mice (6 weeks of age, gender-matched) were obtained by mating heterozygous male and female mice originating from F1 heterozygotes.

#### Breeding and housing

All mice were bred and maintained under specific and opportunistic pathogen-free (SPF) conditions at 22°C ± 2°C, under a 12-h light–dark cycle, at the South Australian Health and Medical Research Institute (SAHMRI). All mice were housed in individually ventilated cages, fed an identical diet (Teklad Global 18% Rodent Protein Diet, Envigo, Huntingdon, UK), maintained under the Federation of European Laboratory Animal Science Associations (FELASA) standards, and routinely screened using an SNP genotyping panel.

**Figure 1:**
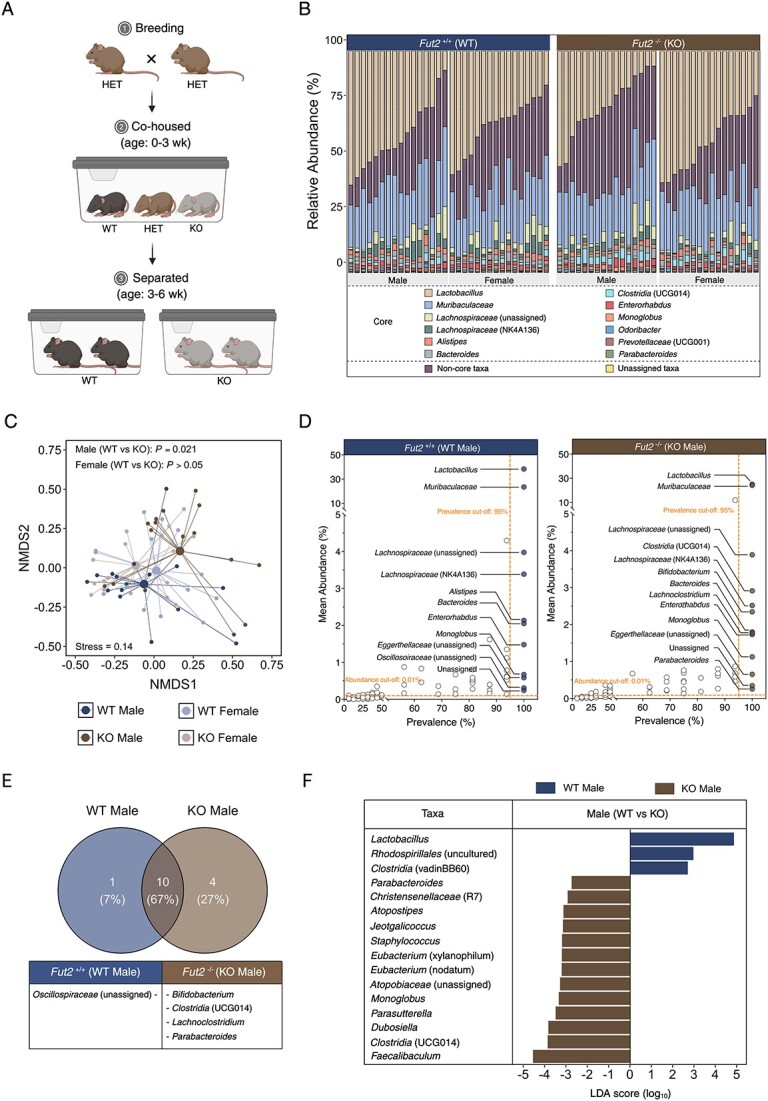
*Fut2* affects the faecal microbiota of male mice. (A) Breeding and cohousing design. *Fut2*^HET^ mice were bred to produce *Fut2*^WT^ and *Fut2*^KO^ littermates. Genotypes were co-housed until weaning (~3 weeks), where mice were separated by sex and *Fut2* genotype until 6 weeks of age. (B) Taxa bar plot of the faecal microbiota of *Fut2*^WT^ and *Fut2*^KO^ mice, also stratified by sex. (C) NMDS plot of faecal microbiota showing significant separation between *Fut2*^WT^ and *Fut2*^KO^ male mice but not *Fut2*^WT^ and *Fut2*^KO^ female mice. Significance: Permutational multivariate ANOVA. (D) the prevalence and mean relative abundance of faecal taxa in *Fut2*^WT^ and *Fut2*^KO^ male mice, highlighting the “core” taxa present in ≥95% of mice and at a mean relative abundance of ≥0.01%. (E) Venn diagram of core taxa shared or different between *Fut2*^WT^ and *Fut2*^KO^ male mice. (F) Faecal taxa significantly different between *Fut2*^WT^ and *Fut2*^KO^ male mice. Significance: LEfSe.

Heterozygous × heterozygous breeding was performed to allow for *Fut2*^KO^ and *Fut2*^WT^ littermates, while also standardizing effects of *Fut2* that occur through vertical transmission. *Fut2*^KO^, *Fut2*^HET^, and *Fut2*^WT^ littermates were cohoused from birth until weaning (~3 weeks), where they were genotyped by PCR amplicon melt curve using primers targeting the outer and inner regions of the *Fut2* gene. *Fut2*^KO^ and *Fut2*^WT^ mice separated into cages after weaning based on sex and *Fut2* genotype (Fig. 1A). No experiments were performed on *Fut2*^HET^ mice. In all experiments, 6-week old, age- and sex-matched mice were used. Each experimental group consisted of at least four cages to control for cage effects. Given the heterogeneous nature of the gut microbiome, each mouse was considered as a biological replicate rather than a technical replicate, even within cohoused littermates.

#### Antibiotic treatment

A cocktail of ampicillin (1 g/L, Sigma-Aldrich) and neomycin (0.5 g/L, Sigma-Aldrich) was provided to mice via drinking water for 7 days. Water bottle volume and mouse weight were monitored to assess water intake. Antibiotic activity was confirmed by qPCR targeting 16S rRNA gene (Supplementary Table S2) of faecal samples at day 7.

#### Probiotic supplementation

Mice received 5 × 10^7^ colony-forming units (CFU)/g of mouse of either *B. infantis, B. bifidum*, or *B. breve* daily for 5 days via oral gavage. A starting gavage concentration of 5 × 10^9^ CFU/ml in phosphate-buffered saline (PBS) was prepared daily from fresh overnight cultures. The dose was selected based on previous reports of safety, persistence, and immune modulation capability [22, 23].

### Bacterial strains

*Bifidobacterium bifidum* JCM 1255 (ATCC equivalent: 29521), *B. longum subspecies infantis* JCM 1222 (ATCC equivalent: 15697), and *B. breve* JCM 1192 (ATCC equivalent: 15700) were obtained from the Japan Collection of Microorganisms (JCM; RIKEN, Saitama, Japan). Utilization of α(1,2)-fucosylated glycans was assessed by *in vitro* culture in mBasal media with/without supplementation of 5% w/v 2′-fucosyllactose (Layer Origin, Ithaca, NY, USA) (Supplementary Fig. S2). This was confirmed by existing literature [24, 25] and the Carbohydrate-Active enZYmes (CAZy) database [26] showing that *B. bifidum* JCM 1255 encodes external GH29 and GH95 α-1,2-L-fucosidases, but does not consume fucose as a carbon source [27], *Bifidobacterium infantis* JCM 1222 encodes intracellular GH29, GH95, and GH151 α-1,2-L-fucosidases along with glycan transporters, and *B. breve* JCM 1192 encodes only the GH95 family.

### Faecal and tissue collection

At least two faecal pellets were collected from separated mice at the beginning of the light phase, unless specified otherwise. For intestinal tissue collection, mice were sacrificed by CO_2_ asphyxiation and laparotomy was immediately performed using a vertical midline incision. Once the digestive tract was exposed, separate dissection tools were used to dissect tissue into four parts: the proximal small intestine; distal small intestine; caecum; and large intestine. For small and large intestine tissue segments, the luminal content was collected by instilling sterile PBS using a syringe barrel and the flushed mucosal tissue was collected into separate tubes. All collected faecal samples, organs, and luminal contents were immediately frozen on dry ice and stored at −80°C until further processing.

### DNA extraction

#### DNA extraction of faecal samples

Faecal pellets were weighed, and 25-mg samples (±10 mg) were resuspended in 300 μl of cold PBS (pH 7.2) by vortexing and pelleted by centrifugation at 10 000 × *g* for 10 min at 4°C. Microbial DNA was extracted from faecal samples using the PowerLyzer PowerSoil DNA Isolation Kit (Qiagen, Hilden, Germany) according to the manufacturer’s instructions as described previously [28].

#### DNA extraction on mucosal tissue samples

Mucosal tissue from the proximal small intestine, distal small intestine, and large intestine were semi defrosted, and 3 cm was removed from the tissue centre using sterile scalpel. The dissected tissues were cut open longitudinally and mixed with 750-μl PowerSoil bead solution and 60 μl solution C1 in a PowerSoil bead tube. The bead tube was then incubated at 65°C for 10 min prior to bead beating. The subsequent DNA isolation was performed using the PowerLyzer PowerSoil DNA Isolation Kit (Qiagen, Hilden, Germany) according to the manufacturer’s instructions as described previously [28].

### 16S rRNA gene amplicon sequencing and bioinformatic processing

Amplicon libraries of the V4 hypervariable region for 16S rRNA gene were prepared from DNA extracts using modified universal bacterial primer pairs 515F and 806R [29]. Amplicon libraries were indexed, cleaned, and sequenced according to the 16S Metagenomic Sequencing Library Preparation protocol. Paired-end sequencing was performed using MiSeq Reagent Kit v3 (600-cycle kit) (Illumina) on a MiSeq System (Illumina), at the South Australian Genomics Centre (SAGC). Paired-end 16S rRNA gene sequence reads were analysed using QIIME2 version 2021.11.0 [30]. Briefly, de-noising was performed on de-multiplexed sequences using the DADA2 plugin [31], resulting in a mean read depth of 15 563 ± 2719 for stool and 6941 ± 4503 for tissue. Taxonomic classification of amplicon sequence variants (ASVs) was performed based on the V4 hypervariable region of the SILVA 16S rRNA gene reference database (version 138) at 99% similarity [32]. Sufficient coverage at this depth is confirmed by the rarefaction curve, which reached an asymptote. Sequence data have been deposited in the National Center for Biotechnology Information Sequence Read Archive (NCBI SRA) under accession number PRJNA1011386.

### Microbiota characterization

The taxonomic relative abundance at the genus level was used to generate alpha diversity (within-group) and beta diversity (between-group) measures. Alpha diversity metrics (observed ASVs, Pielou’s evenness, Shannon diversity, and Faith’s phylogenetic diversity) were obtained from QIIME2 at sampling depth of 9883 reads (faecal samples) and 662 reads (mucosal tissue samples). The Bray–Curtis dissimilarity index was calculated to compare microbiome similarity between groups (beta diversity), using square-root-transformed species relative abundance data using the “vegan” package in R. Nonmetric multidimensional scaling (nMDS) for all beta diversity measures were generated using the “vegan” package in R. Core taxa were defined as those present in >95% of samples, with a mean relative abundance of >0.01%. Identification of taxa with α-1,2-L-fucosidases capability was determined by comparing the genus-level taxonomic classification to genomes identified by CAZy [26] as carrying either the GH29, GH95, or GH151 enzyme families.

### Quantification of *Bifidobacterium* species and total bacterial load

We investigated the extent to which 16S amplicon sequencing could discriminate between different *Bifidobacterium* species. As expected, level 7 resolution (species-level output) was unable to differentiate bifidobacterial strains, reflecting a well-recognized limitation of this approach. Given that, quantification of total bacterial load, *B. breve*, *B. infantis*, and *B. bifidum* was performed by SYBR Green-based qPCR assays (Supplementary Table S2). For all qPCR assays, 1 μl of DNA template was combined with 0.7 μl of 10 μM forward primer, 0.7 μl of 10 μM reverse primer, 17.5 μl of 2 × SYBR Green (Applied Biosystems, Waltham, MA, USA), and 15.1 μl nuclease-free water. All samples were run in triplicate (10 μl each replicate). Gene copy quantification was performed using a standard curve generated from a known concentration of a pure colony control. Any sample with a cycle threshold (CT) ≥40 cycles was defined as 40 (limit of detection).

### Culture of *Bifidobacterium* strains

All *Bifidobacterium* strains used in this study were cultured in de Man, Rogosa, and Sharpe (MRS) broth or agar (Becton Dickinson, Franklin Lakes, NJ, USA) supplemented with 0.34% (w/v) sodium ascorbate and 0.02% (w/v) cysteine-HCl (MRS-CS) and were grown under anaerobic conditions (75% N2, 20% CO2, 5% H2, Coy Laboratory Products, Grass Lake, MI, USA) at 37°C. The growth of *Bifidobacterium* was measured by optical density (OD_600_) using multimode plate reader (PerkinElmer, Waltham, MA, USA).

### *In vitro* glycan utilization assay

Faecal pellets from untreated wild type (WT) mice were incubated in mBasal media (mBasal; 10 g/L Trypton, 2 g/L yeast extract, 5 g/L NaCl, 0.2 g/L magnesium sulphate, 2 g/L dipotassium hydrogen phosphate, pH 6.4) with/without supplementation of 5% w/v of 2′-fucosyllactose (Layer Origin, Ithaca, NY, USA) at 37°C under strict anaerobic conditions (75% N2, 20% CO2, 5% H2, Coy Laboratory Products, Grass Lake, MI, USA). Bacterial biomass was measured by optical density (OD_600_) using multimode plate reader (PerkinElmer, Waltham, MA, USA). Bacterial colonies cultured from faecal samples were identified by matrix-assisted laser desorption/ionization time-of-flight (MALDI-TOF) (Billerica, MA, United States), as described previously [33].

### Statistical analysis

Experimental mice were randomly assigned to different treatment groups. The investigators were not blinded to the experimental groups. No outliers have been removed from any of the data presented. All data analyses were performed using either R (R Foundation for Statistical Computing; version 4.1.0) or GraphPad Prism software (GraphPad Software, Inc.; version 9.00). For parametric data, unpaired Student’s *t* test was used to compare data between two unpaired groups; one-way ANOVA was used to compare data among three or more unpaired groups. For nonparametric data, the Mann–Whitney U test was used to compare data between two unpaired groups; the Kruskal–Wallis test was used to compared data among three or more unpaired groups. Differences in Bray–Curtis dissimilarity between groups was performed by permutational multivariate ANOVA (PERMANOVA) and pairwise PERMANOVA, using the “adonis” package in R, with 9999. Linear discriminant analysis effect size (LEfSe) was applied to identify the abundant taxa in each site, using default parameters [34]. The area under the curve (AUC) was calculated for *in vitro* growth experiments (using OD_600_ values) and bifidobacterial persistence in mice (using copies/ng faecal DNA). The log-rank test was employed to compare survival time differences based on bacterial qPCR detection. One-tailed tests were used where differences between groups were hypothesized to be in a single direction. Statistical outcomes with *P* value <.05 were considered statistically significant. The core taxa plot was generated using GraphPad Prism; other data were visualized using R.

## Results

### *Fut2* shapes the faecal microbiota

Assessment of the faecal microbiota between SPF *Fut2*^WT^ and *Fut2*^KO^ littermates was performed at 6 weeks of age in both male and female mice (Fig. 1A). Faecal microbiota composition (Fig. 1B) differed significantly between *Fut2*^WT^ and *Fut2*^KO^ littermates (PERMANOVA: *R*^2^ = 0.028; *P =* .028, Fig. 1C) when male and female mice were assessed together. However, stratification according to sex identified a greater divergence according to genotype in male mice (PERMANOVA: *R*^2^ = 0.12; *P =* .021) compared to female mice (PERMANOVA: *R*^2^ = 0.037; *P =* .38, Fig. 1C). Exploration of this sex effect identified a significant interaction between sex and *Fut2* genotype (PERMANOVA: R^2^ = 0.11; *P =* .0068, Supplementary Table S3). These findings were unchanged after adjustment for cage effects (Supplementary Table S3). Given the interaction between sex and genotype, all subsequent experiments involved male mice only.

Potential relationships between secretor status and microbiota characteristics were then explored. While bacterial alpha diversity measures did not differ substantially between differed between *Fut2*^WT^ and *Fut2*^KO^ groups (Supplementary Fig. S3), the membership of core microbiota (taxa present in ≥95% of samples at ≥0.01%) did (Fig. 1D). Specifically, *Oscillospiraceae* (unassigned) were exclusively core in *Fut2*^WT^, whereas *Bifidobacterium*, Clostridia (UCG014), *Lachnoclostridium*, and *Parabacteroides* were exclusively core in *Fut2*^KO^ mice (Fig. 1E). Three bacterial genera had a significantly higher relative abundance in *Fut2*^WT^ mice, whereas 13 were more prevalent in *Fut2*^KO^ mice (Fig. 1F). Of these, *Lactobacillus,* a genus with GH29 and GH151 that comprise α-1,2-L-fucosidases (Supplementary Table S4), was more abundant in *Fut2*^WT^ (LDA score [log_10_] = 4.88). Together, these findings support previous reports of a relationship between *Fut2* and faecal microbiome characteristics.

### Distal small intestine microbiota influenced by *Fut2*

**Figure 2:**
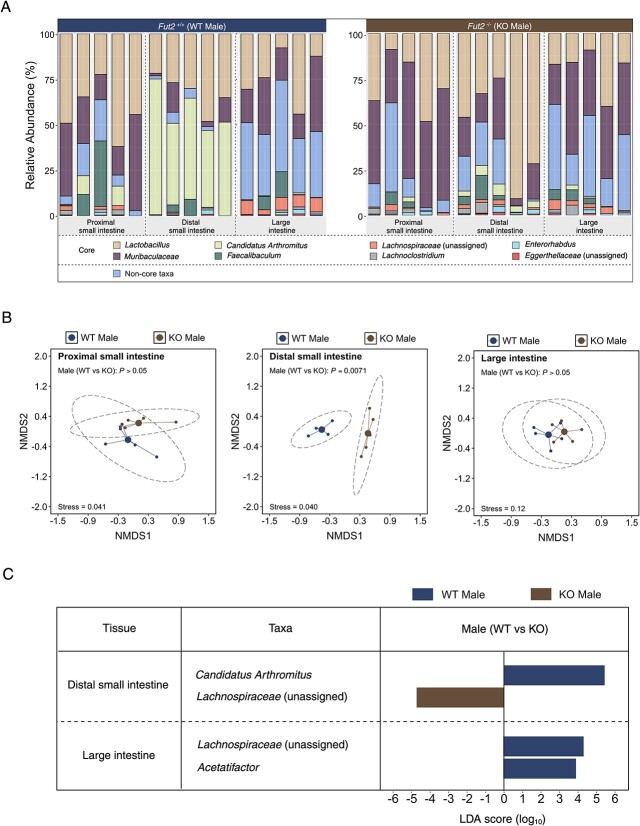
*Fut2* affects the intestinal microbiota. (A) Taxa bar plot of the intestinal mucosal microbiota of *Fut2*^WT^ and *Fut2*^KO^ mice. (B) NMDS plots of *Fut2*^WT^ and *Fut2*^KO^ intestinal mucosal microbiota. Significance: Permutational multivariate ANOVA. (C) Taxa significantly different between *Fut2*^WT^ and *Fut2*^KO^ intestinal mucosal tissue. Significance: LEfSe. No taxa differed between *Fut2*^WT^ and *Fut2*^KO^ proximal small intestine.

As *Fut2* is differentially expressed throughout the gastrointestinal tract [35], we then compared microbiota characteristics in tissue from the proximal small intestine, the distal small intestine, and the large intestine between *Fut2*^WT^ and *Fut2*^KO^ mice (Fig. 2A). Microbiota composition differed significantly between *Fut2*^WT^ and *Fut2*^KO^ groups in the distal small intestine (PERMANOVA: *R*^2^ = 0.54; *P =* .0071, Fig. 2B), coinciding with inducible *Fut2* expression, but not in the proximal small intestine (PERMANOVA: *R*^2^ = 0.19; *P =* .13, Fig. 2B) or the large intestine (PERMANOVA: *R*^2^ = 0.16; *P =* .18, Fig. 2B). Taxonomic assessment identified that *Candidatus* Arthromitus (LDA score [log_10_] = 5.43) was more abundant in the distal small intestine in *Fut2*^WT^ compared to *Fut2*^KO^ mice, whereas *Lachnospiraceae* (unassigned) and *Acetatifactor* were more abundant in in the large intestine of *Fut2*^WT^ compared to *Fut2*^KO^ mice (LDA score [log_10_] = 4.35, and LDA score [log_10_] = 3.93, respectively) (Fig. 2C).

### *Fut2*–microbiota relationships can be recapitulated *in vitro* through glycan exposure

**Figure 3:**
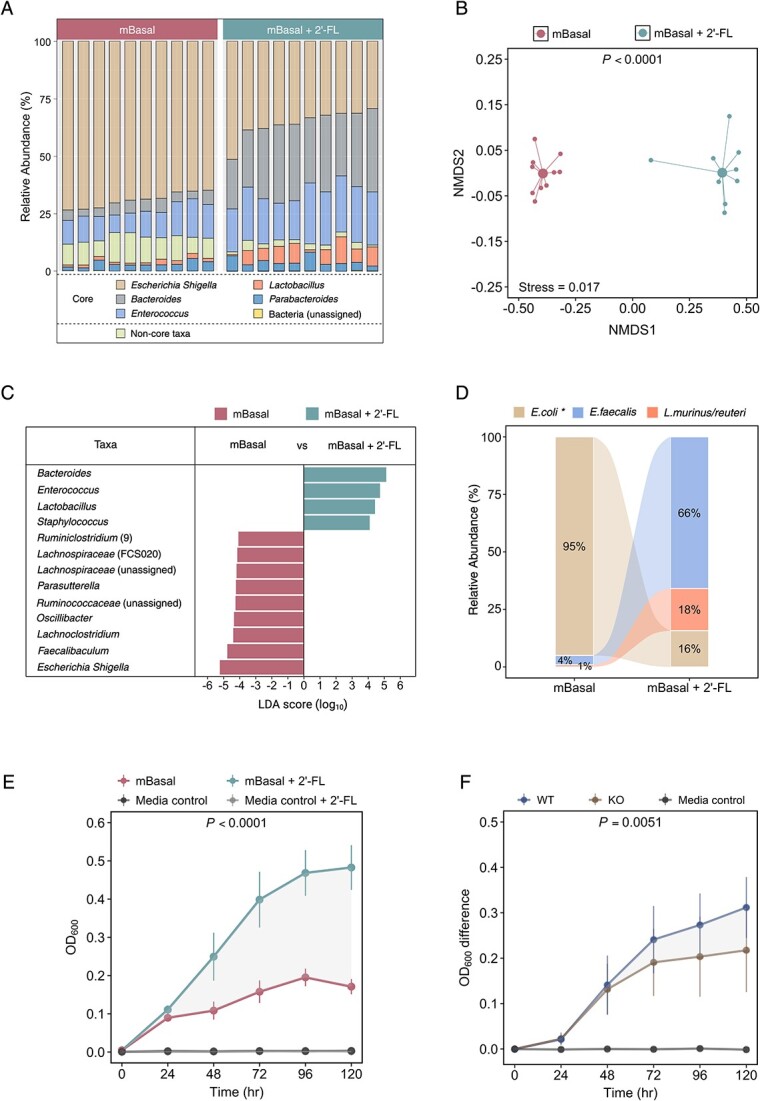
2′-Fucosyllactose modifies the faecal microbiota in vitro and enhances growth of α(1,2)-fucosylated glycan-utilizing bacteria. (A) Taxa bar plot of the faecal microbiota of *Fut2*^WT^ mice following anaerobic growth either with or without 2′-fucosyllactose (2′-FL). (B) NMDS plot of faecal microbiota following anaerobic growth with or without 2′-FL. significance: Permutational multivariate ANOVA. (C) Taxa significantly different between *Fut2*^WT^ faecal samples following anaerobic growth with or without 2′-FL. Significance: LEfSe. (D) Comparison of colonies relative abundance of identified colonies following anaerobic growth with or without 2′-FL. *E. coli or *Shigella.* (E) OD following *Fut2*^WT^ faecal bacteria cultured with or without 2′-FL. (F) OD of *Fut2*^WT^ or *Fut2*^KO^ faecal bacteria following growth with 2'-FL. OD normalized to growth in media without 2'-FL.

To further investigate the relationship between α(1,2)-fucosylated glycans and intestinal microbiology, faecal homogenate from *Fut2*^WT^ mice was grown in a mBasal media with or without the α(1,2)-fucosylated glycan, 2'-FL. Microbiota assessment following *in vitro* culture (Fig. 3A) confirmed the findings of the *in vivo* faecal microbiota analysis, with a significant difference in Bray–Curtis similarity between faecal cultures with and without 2'-FL (PERMANOVA: *R*^2^ = 0.90; *P <* .0001, Fig. 3B). This difference was marked by an enrichment of glycan-utilizing genera (*Bacteroides*, *Enterococcus*, *Lactobacillus*), with compensatory decreases in the relative abundance of other taxa (Fig. 3C).

Analysis involving inoculation of solid basal media, either alone or supplemented with 2'-FL, with faecal homogenate from *Fut2^WT^* mice further supported these findings. Specifically, proportional colony counts (Supplementary Fig. S4, Supplementary Table S5) showed 2'-FL led to enrichment of *Enterococcus faecalis* (66% with 2'-FL vs 4% without 2'-FL) and *Lactobacillus murinus/reuteri* (18% with 2'-FL vs 1% without 2'-FL), with a corresponding decrease in *E. coli* (16% with 2'-FL vs 95% without 2'-FL) (Fig. 3D). Enrichment of bacteria with 2'-FL supplementation was further confirmed by increased growth rate and bacterial density (median AUC_[mBasal]_ = 15.1 [IQR = 14.3, 16.5]; AUC_[mBasal + 2'-FL]_ = 35.6 [33.3, 38.7]; *P <* 0.0001, Fig. 3E).

We investigated whether differences in microbiota composition between *Fut2*^WT^ and *Fut2*^KO^ mice reflected selection for bacterial populations able to utilize α(1,2)-fucosylated glycans for growth in *Fut2^WT^* animals. Faecal homogenates from *Fut2*^WT^ and *Fut2*^KO^ mice were used to inoculate mBasal media with or without 2'-FL supplementation. The increase in bacterial density between 2'-FL supplemented media and media alone was significantly greater when faecal homogenates were derived from *Fut2*^WT^ compared to *Fut2*^KO^ mice (median AUC_[WT]_ = 20.5 [IQR = 16.6, 23.5]; AUC_[KO]_ = 16.7 [13.1, 19.7]; *P =* 0.0051, Fig. 3F), consistent with a greater abundance of glycan-utilizing bacteria.

### Probiotic bifidobacterial species differentially colonize *Fut2*^WT^ and *Fut2*^KO^ mice

**Figure 4:**
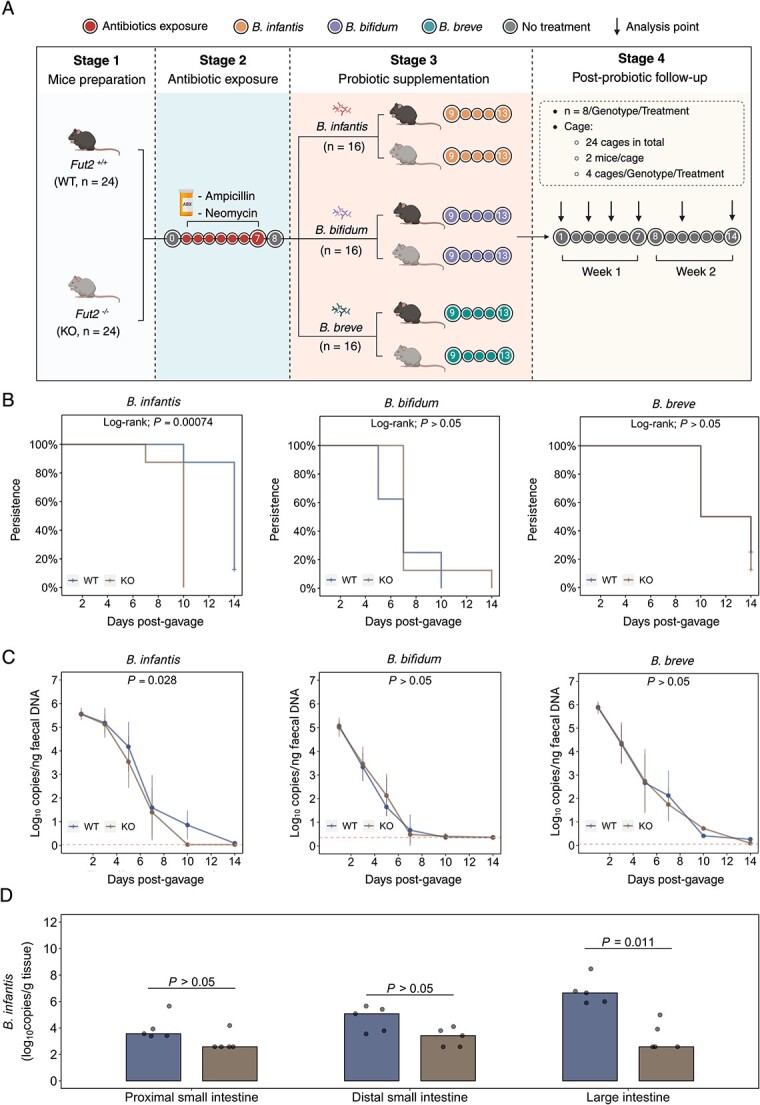
*Bifidobacterium infantis*, but not *B. breve* or *B. bifidum,* persists longer in *Fut2*^WT^ mice following antibiotic pre-exposure. (A) Experimental design. (B) Persistence of detectable *Bifidobacterium* species in stool following antibiotic pre-exposure. Significance: log-rank test. (C) Bacterial copies of *Bifidobacterium* species in stool following antibiotic pre-exposure. Significance: T-test of area under the curve. (D) Bacterial copies of *B. infantis* in intestinal tissue mucosa following antibiotic pre-exposure. Significance: Mann–Whitney U test.

Based on the relationship between host glycan production and gut microbiota composition, we hypothesized that the colonization dynamics (abundance and persistence) of bifidobacterial probiotic species, introduced following antibiotic depletion, would differ between *Fut2*^WT^ and *Fut2*^KO^ recipients (Fig. 4A). We utilized strains of three *Bifidobacterium* species that interact with glycan in different ways: *B. infantis*, an intracellular α(1,2)-fucosylated glycan-utilizer, *B. bifidum*, an extra-cellular α(1,2)-fucosylated glycan utiliser, and *B. breve*, a species that does not utilize α(1,2)-fucosylated glycans (characteristics that were confirmed by *in vitro* growth assays, Supplementary Fig. S2).

The persistence of species that do not internalize and degrade α(1,2)-fucosylated glycans (*B. bifidum* and *B. breve*) did not differ substantially when introduced to *Fut2*^WT^ or *Fut2*^KO^ mice (Fig. 4B and C). In contrast, the abundance and persistence of *B. infantis*, an intracellular glycan utilizer, differed significantly according to the genotype of the recipient (Fig. 4B and C). Ten days postgavage, *B. infantis* was detected in seven out of eight *Fut2*^WT^ mice but zero out of eight *Fut2*^KO^ mice (Log-rank test: chi-q = 11.4; *P =* .00074, Fig. 4B). The overall abundance of *B. infantis* was also significantly higher in *Fut2*^WT^ compared to *Fut2*^KO^ mice in the following 14 days postgavage (mean AUC_[WT]_ = 31.1 [SD = 4.1]; AUC_[KO]_ = 26.2 [5.2]; *P =* .028, Fig. 4C).

A difference in *B. infantis* persistence was also evident in intestinal tissue assessed 5 days postgavage. *Bifidobacterium infantis* was significantly more abundant in *Fut2*^WT^ large intestine (median *Fut2*^WT^ = log_10_ 6.7 copies/g tissue [IQR = log_10_ 6.0, log_10_ 6.8]; *Fut2*^KO^ = log_10_ 2.6 [IQR = log_10_ 2.6, log_10_ 3.9]; *P =* .011) and numerically, though not significantly, more abundant in *Fut2*^WT^ proximal (*P =* 0.085) and distal small intestine (*P =* .094) (Fig. 4D).

### Prior antibiotic exposure profoundly affects the *Fut2*–probiotic relationship

**Figure 5:**
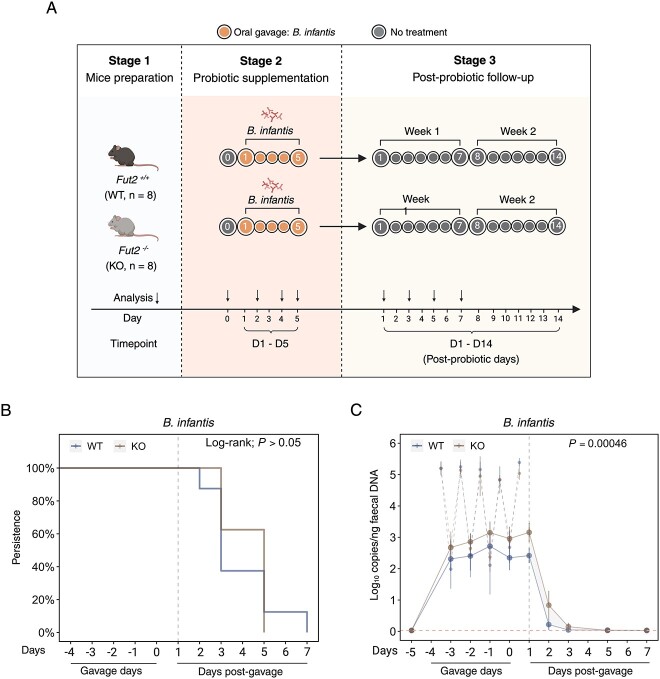
*Bifidobacterium infantis* persists longer in *Fut2*^KO^ mice without antibiotic pretreatment. (A) Experimental design. (B) Persistence of gavaged *B. infantis* in stool without antibiotic pre-treatment. Significance: Log-rank test. (C) Bacterial copies of *B. infantis* in stool without antibiotic pre-treatment. Significance: T-test of area under the curve.

To test whether the relationship between *Fut2* genotype and probiotic strain characteristics were independent of antibiotic exposure, we supplemented nonantibiotic-exposed mice with *B. infantis* (Fig. 5A). No significant difference in *B. infantis* persistence postgavage was observed between *Fut2*^WT^ and *Fut2*^KO^ mice (Fig. 5B), and *B. infantis* was not detectable in intestinal tissue from either *Fut2*^WT^ or *Fut2*^KO^ mice at day 5 postgavage (Supplementary Fig. S5A). However, analysis of *B. infantis* abundance following gavage (based on AUC) revealed an effect that was opposite to that observed in antibiotic exposed mice, with *B. infantis* significantly higher in *Fut2*^KO^ mice compared to *Fut2*^WT^ (mean AUC_[WT]_ = 3.8 [SD = 0.6]; AUC_[KO]_ = 5.6 [0.90]; *P =* .00046; Fig. 5C).

Faecal levels of *Bifidobacterium* probiotics were assessed during the instillation period (samples collected 2 h prior to gavage and 6 h after gavage). Levels in postgavage samples (6 h) did not differ between *Fut2*^WT^ and *Fut2*^KO^ mice, consistent with the instillation of equal probiotic loads. However, at 22 h postgavage (2 h prior to gavage), levels in *Fut2*^KO^ mice were significantly higher than in *Fut2*^WT^ mice (*P <* .0001; Supplementary Fig. S5B). No cumulative effect was observed with repeat gavage and the decline in probiotic levels after 22 h was comparable to that observed in the 24 h postcessation of installation.

## Discussion

Although probiotics have shown great potential in modifying host–microbiome interactions [36], their actual performance has been disappointing in many clinical contexts [37]. Previous studies have investigated the reasons for this underperformance, relating to cohort-level effects and variation in response between individuals. Mode of delivery and dose have both been shown to contribute to overall efficacy [38, 39], whereas the habitual diet of the recipient, particularly fibre intake, is an important determinant of probiotic response [40]. The influence of factors that shape the gut microbiome on the abundance and persistence of probiotics is unsurprising [41, 42], given the ability of resident gut microbiota to competitively exclude introduced populations [10]. Indeed, exposure to antibiotics, a factor that greatly impacts the gut microbiome, has been shown to considerably influence probiotic effects at a microbiological level [9, 43]. However, while common *FUT2* genetic variants are known to help shape intestinal microbiology [16, 17], the effect of secretor status on probiotics had not been described.

Our study highlights several important points in relation to interindividual variance in intestinal microbiology and probiotic efficacy. With 20% of the global population also homozygous for a nonfunctional *FUT2* gene [15], our findings suggest that these “non-secretor” individuals will also experience different probiotic population dynamics compared to “secretor” individuals, if the probiotic taken contains one of the many bacterial species able to utilize α(1,2)-fucosylated glycans (H antigens). In our study, this was reflected in the significantly greater transience of *B. infantis* in the faecal and intestinal microbiome of nonsecretor (*Fut2*^KO^) mice compared with secretor mice following antibiotic exposure.

Bifidobacterial species that are commonly used as probiotics are relatively close phylogenetically but differ in their ability to use glycans, even at a strain level [44]. We showed that neither *B. breve* (JCM 1192) nor *B. bifidum* (JCM 1255) differed in their abundance or persistence between secretor and nonsecretor animals. In contrast, *B. infantis* (JCM 1222) persisted for significantly longer and showed a significantly higher abundance in secretor mice compared to nonsecretor mice. This finding likely reflects differences in H antigen hydrolysis and catabolism capacities between species when administered as a probiotic. For example, *B. infantis* encodes GH29, GH95, and GH151 family intracellular α-1,2-L-fucosidases, along with fucose transporters to facilitate internalization [44]. While independent hydrolysis and catabolism of mucin-bound H antigens by *B. infantis* are not hypothesized [25], cross-feeding by organisms with extracellular α-1,2-L-fucosidases is likely, even following antibiotic supplementation [45]. In contrast, *B. bifidum*, while expressing extracellular GH29 and GH95 α-1,2-L-fucosidases, does not consume fucose to facilitate growth [27]. Finally, *B. breve* encodes a separate GH95 intracellular α-1,2-L-fucosidase along with fucose transporters. While this species is capable of utilizing the H antigen with support from cross-feeding [46], these findings suggest reduced persistence compared with *B. infantis*, when administered as a probiotic.

We found that antibiotic exposure influenced the persistence of probiotics in a secretor status–dependent manner. In the absence of microbiota depletion through antibiotic exposure, it would be expected that other commensal bacteria would utilize available glycans within the secretor gut. Moreover, such strains are highly adapted to an individual’s gut environment, making them likely to outcompete any exogenous glycan utilizers that are introduced. When we explored this directly, we found that in the absence of a prior period of antibiotic exposure, the higher levels and greater persistence of *B. infantis* in secretors was inverted, with these *B. infantis* being significantly higher in nonsecretor mice. These findings likely reflect the competitive exclusion of H antigen-utilizing probiotics in the secretor gut and highlight the importance of considering the ecological context in relation to probiotic impact.

It should be noted that our antibiotic mix contained a cocktail of ampicillin and neomycin, designed to deplete a wide range of bacteria. While most *Bifidobacterium* strains are resistant to neomycin, the tested strains are sensitive to ampicillin [47]. We designed the experiment so that gavage with *Bifidobacterium* was immediately after ceasing antibiotic depletion to maximize colonization without competition from other bacteria. It is possible that residual antibiotics in the intestine deplete *Bifidobacterium* over the first days of gavage. For this reason, we performed gavage for 5 days, a time period that extends beyond the activity spectrum of the administered antibiotics. Such an antibiotic combination is common for mouse models [48, 49], as well as empiric for suspected sepsis in humans [50].

While this study was performed in mice, the effect of secretor status on bifidobacterium supplementation has important implications for probiotic strategies in humans. It is crucial to consider individual host traits and recent antibiotic exposure when designing a probiotic intervention [51]. The findings here suggest that the 20% of the population who are nonsecretors may have poorer persistence of H antigen utilizing probiotics, such as *B. infantis,* compared to secretors following antibiotic exposure. Conversely, in the absence of recent antibiotic exposure, higher levels of microbial niche occupancy in secretors may hamper *B. infantis* persistence compared to nonsecretors. An individualised supplementation with prebiotics may have potential as a means to optimize probiotic uptake in nonsecretors. For example, previous studies have shown that supplementation with human milk oligosaccharides can enhance *B. infantis* engraftment [20], with successful supplementation shown to reduce intestinal inflammation in infants [52]. Investigating additional α(1,2)-fucosylated glycans, given as prebiotics, may lead to improved outcomes of *B. infantis* supplementation in nonsecretor individuals.

Determining the impact of secretor status on other species commonly considered beneficial and marketed as probiotics is challenging due to their broad range of carbohydrate utilization capabilities [53]. For instance, *Akkermansia muciniphila*, a mucin-degrading species, has been associated with a reduced risk of chronic inflammatory diseases in humans and mice [54], and is a potential target for probiotic development [51]. However, its utilization of mucin glycoproteins, including the α(1,2)-fucosylated glycan, 2′-fucosyllactose [53], suggests that it may also be affected by secretor status. Although *Akkermansia* was not detected in the mice of this study, we found that *Candidatus* Arthromitus, another genus associated with immune modulation [55], was enriched in the distal small intestinal mucosal tissue of secretor mice. Genome annotation of *Candidatus* Arthromitus has indicated other fucose utilization capabilities [56], indicating that the functional *FUT2* gene may promote colonization by this species.

Our experiments involved SPF mice that were obtained through heterozygous mating. Such breeding was essential to allow comparison of *Fut2*^WT^ and *Fut2*^KO^ littermates from a maternal secretor lineage. The findings from this study are therefore independent of vertical transmission effects, which are known to influence the microbiome of the offspring [57, 58], and indicate that a change in gut microbiology occurred postweaning. This difference in baseline gut microbiota composition between secretors and nonsecretors was also only evident in male mice. The effect of sex on the relationship between secretor status and the gut microbiome is difficult to explain but may relate to variable intestinal expression of *Fut2*, which can be altered factors such as stress [59]. In addition, independent interactions between sex hormones and the gut microbiome [60] may affect the relationship between *Fut2* and the gut microbiome.

We acknowledge the importance of considering blood antigens/ABO phenotypes in interpreting the influence of *FUT2* gene on the gut microbiome, as indicated by recent studies [61, 62]. Indeed, in humans, *FUT2* is responsible for the generation of the H antigen, which can be further modified to give the OLewis^b^, ALewis^b^, or BLewis^b^ antigens [63]. Each of these glycans can modulate the competitive advantage of particular microbes capable of cleaving the oligosaccharide constituents. In the absence of *FUT2*, these Lewis^b^ antigens are not displayed, leading to a Lewis^a^ antigen. While our study did not address these blood type variations, it should be noted that even in humans, a secretor O blood group and a nonsecretor O blood group are not the same. The impact of this on the gut microbiome is evidenced by studies reporting an association between H antigen concentrations and gut microbiome characteristics [58].

Our study demonstrates a *Fut2*-dependent genetic determinant for interindividual response to probiotic supplementation, which is affected by antibiotic exposure and glycan utilization capabilities of the probiotic strain. With prior antibiotic exposure, *Fut2* functionality was associated with increased persistence of *B. infantis*, consistent with its ability to utilize the H antigen. However, without antibiotic exposure, *Fut2* functionality was associated with lower abundance of *B. infantis,* relating to difference in baseline microbiology and niche space occupation.

## Supplementary Material

Supplementary_information_Final_wrae107

Supplementary_Table_S4_wrae107

## Data Availability

All 16S rRNA gene sequencing data have been deposited to the NCBI SRA and are available under accession number PRJNA1011386. All qPCR data are available through FigShare (https://figshare.com/s/9fa55ea8b65304d9f722). Data processing, statistical analysis, and visualization is available through GitHub (https://github.com/Yiming-Wang-1992).
