# Supplementary material for: Intestinal persistence of Bifidobacterium infantis is determined by interaction of host genetics and antibiotic exposure
Source: ISME J. 2024 Jun 19;18(1):wrae107. doi: 10.1093/ismejo/wrae107 (PMC11214156; doi:10.1093/ismejo/wrae107)
Supplement: Supplementary_information_Final_wrae107 [file supplementary_information_final_wrae107.docx]

Supporting Information for

**Intestinal persistence of *Bifidobacterium infantis* is determined by interaction of host genetics and antibiotic exposure**

Running title: *FUT2* impacts probiotic persistence

Yiming Wang, Jocelyn M Choo, Alyson C Richard, Lito E Papanicolas, Steve L Wesselingh, Steven L Taylor^#^, Geraint B Rogers^#*^

^#^ These authors share senior authorship.

**^*^ Correspondence:**

Professor Geraint Rogers

Director, Microbiome & Host Health, South Australian Health and Medical Research Institute, North Terrace, Adelaide, SA 5001, Australia.

**Email:** [geraint.rogers@sahmri.com](mailto:geraint.rogers@sahmri.com)

**Phone:** +61 (0)403 558251

**This PDF file includes:**

- Tables S1 - S4
- Figures S1 - S5

**Table S1: Reagent and resource list**

| **REAGENT OR RESOURCE** | **SOURCE** | **IDENTIFIER** |
| --- | --- | --- |
| **Antibiotics** | | |
| Ampicillin | Sigma-Aldrich | N1876 |
| Neomycin | Sigma-Aldrich | A1593 |
| **Bacterial and Virus strains** | | |
| *Bifidobacterium bifidum* | Japan Collection of Microorganisms | JCM 1255 |
| *Bifidobacterium breve* | Japan Collection of Microorganisms | JCM 1192 |
| *Bifidobacterium longum* subspecies *infantis* | Japan Collection of Microorganisms | JCM 1222 |
| **Chemicals, Peptides, and Recombinant Proteins** | | |
| 2'-Fucosyllactose (98% purity) | Layer Origin, New York, USA | PureHMO |
| Lactobacilli MRS Agar 500G | BD, New Jersey, USA | Cat# 288210 |
| Lactobacilli MRS Broth 500g | BD, New Jersey, USA | Cat# 288130 |
| **Critical Commercial Assays** | | |
| DNeasy PowerLyzer PowerSoil Kit | QIAGEN, Hilden, Germany | Cat# 12855-100 |
| PowerUp SYBR Green Master Mix | Life Technologies | Cat# A25777 |
| MiSeq Reagent Kit v3 (600-cycle) | Illumina | Cat# MS-102-3003 |
| Nextera XT Index Kit v2 Set A | Illumina | Cat# FC-131-2001 |
| NextSeq 500/550 Mid Output Kit v2.5  (300 Cycles) | Illumina | Cat# 20024905 |
| Illumina® DNA Prep, (M) Tagmentation | Illumina | Cat# 20018704 |
| Nextera™ DNA CD Indexes | Illumina | Cat# 20018708 |
| Nextera XT DNA Library Preparation Kit | Illumina | Ca# FC-131-1096 |

| ***Continued*** | | |
| --- | --- | --- |
| **REAGENT OR RESOURCE** | **SOURCE** | **IDENTIFIER** |
| **Experimental Models: Organisms/Strains** | | |
| *Fut2*^+/+^ C57BL/6JSah  (Males and females 6 weeks of age) | SAHMRI, Australia | N/A |
| *Fut2*^-/-^ C57BL/6JSah  (Males and females 6 weeks of age) | SAHMRI, Australia | N/A |
| **Oligonucleotides** | | |
| Primers for genotyping m*Fut2*_KO_F:  5'- GGCACAATGCAGATGATTAG -3' | Designed in this study | N/A |
| Primers for genotyping m*Fut2*_KO_R1:  5'- GCTTCACTATTCGTTGCTGG -3' | Designed in this study | N/A |
| Primers for genotyping m*Fut2*_KO_R2:  5'- AGTTTTCAGGAACAGGAGCC -3' | Designed in this study | N/A |
| 16S V4 Amplicon PCR Forward Primer | [1] | N/A |
| 16S V4 Amplicon PCR Reverse Primer | [1] | N/A |
| Miseq Illumina sequencing Index primer |  |  |
| **Deposited Data** | | |
| Sequence data | NCBI | PRJNA1011386 |
| **Software and Algorithms** | | |
| QIIME 2 version 2021.11.0 | [2] | N/A |
| R studio 4.1.0 | N/A | N/A |
| GraphPad Prism 9.0.0 | GraphPad Software, California, USA | N/A |
| PRIMER 6.1.16 & PERMANOVA+ 1.0.6 | PRIMER-e, Auckland, NZ | N/A |

**Table S2.** qPCR primers and cycling conditions

| **Target** | **Standard DNA** | **Primer sequences** | **Cycling conditions** | **Reference** |
| --- | --- | --- | --- | --- |
| 16S | *E. coli* ATCC | qB331F: TCCTACGGGAGGCAGCAGT  qB797R: GGACTACCAGGGTATCTAATCCTGTT | 40 cycles:  95°C for 15 s  60°C for 1 min | [3] |
| *B. longum sp. infantis* | JCM 1222 | BIN-F: CGCGAGCAAAACAATGGTT  BIN-R: AACGATCGAAACGAACAATAGAGTT | 40 cycles:  95°C for 15 s  60°C for 1 min | [4] |
| *B. bifidum* | JCM 1255 | B_bif-f: CTCCGCAGCCGACCCCGAGGTT  B_bif-r: TGGAAACCTTGCCGGAGGTCAGG | 40 cycles:  94°C for 15 s  64°C for 15 s  72°C for 15 s  83°C for 15 s | [5] |
| *B. breve* | JCM 1192 | BBR-F: GTGGTGGCTTGAGAACTGGATAG  BBR-R: CAAAACGATCGAAACAAACACTAAA | 40 cycles:  95°C for 15 s  60°C for 1 min | [4] |
| *Fut2* | N/A | mFut2KO_F: GGCACAATGCAGATGATTAG  mFut2KO_R1: GCTTCACTATTCGTTGCTGG  mFut2KO_R2: AGTTTTCAGGAACAGGAGCC | 45 cycles:  95°C for 15 s  60°C for 1 min | This study |

**Table S3.** Genotype and sex effect on Microbiome composition

| Model | Covariates | *R*^2^ | *F* value | *P* values* |
| --- | --- | --- | --- | --- |
| PERMANOVA | Genotype | 0.028 | 2.74 | 0.028 |
|  | Sex | 0.022 | 2.14 | 0.072 |
|  | Cage (nested in Genotype + Sex) | 0.49 | 3.05 | 0.0001 |
|  | Residual | 0.46 |  |  |
|  | Total | 1 |  |  |
| Pair-wise PERMANOVA | Genotype x Sex | 0.11 | 2.38 | 0.0068 |
|  | Residual | 0.89 |  |  |
|  | Male (WT vs KO) | 0.12 | 4.28 | 0.021* |
|  | Female (WT vs KO) | 0.037 | 1.14 | 0.38* |
|  | WT (Male vs Female) | 0.040 | 1.24 | 0.38* |
|  | KO (Male vs Female) | 0.12 | 4.02 | 0.021* |

* *P* value after FDR correction

| Taxa | Treatment | Median colony counts  (IQR, log10 CFU/mL) | *P* value |
| --- | --- | --- | --- |
| *Escherichia coli* | mBasal | 8.93 [IQR=8.84, 9.00] | 0.030 |
|  | mBasal + 2ˈ-FL | 7.57 [IQR=7.28, 7.76] |  |
| *Enterococcus faecalis* | mBasal | 7.54 [IQR=7.54, 7.57] | 0.029 |
|  | mBasal + 2ˈ-FL | 8.32 [IQR=8.27, 8.37] |  |
| *Lactobacillus murinus/reuteri* | mBasal | 6.94 [IQR=6.89, 6.96] | 0.030 |
|  | mBasal + 2ˈ-FL | 7.72 [IQR=7.61, 7.84] |  |

**Table S5.** *In vitro* culture of faecal bacteria following by glycan exposure


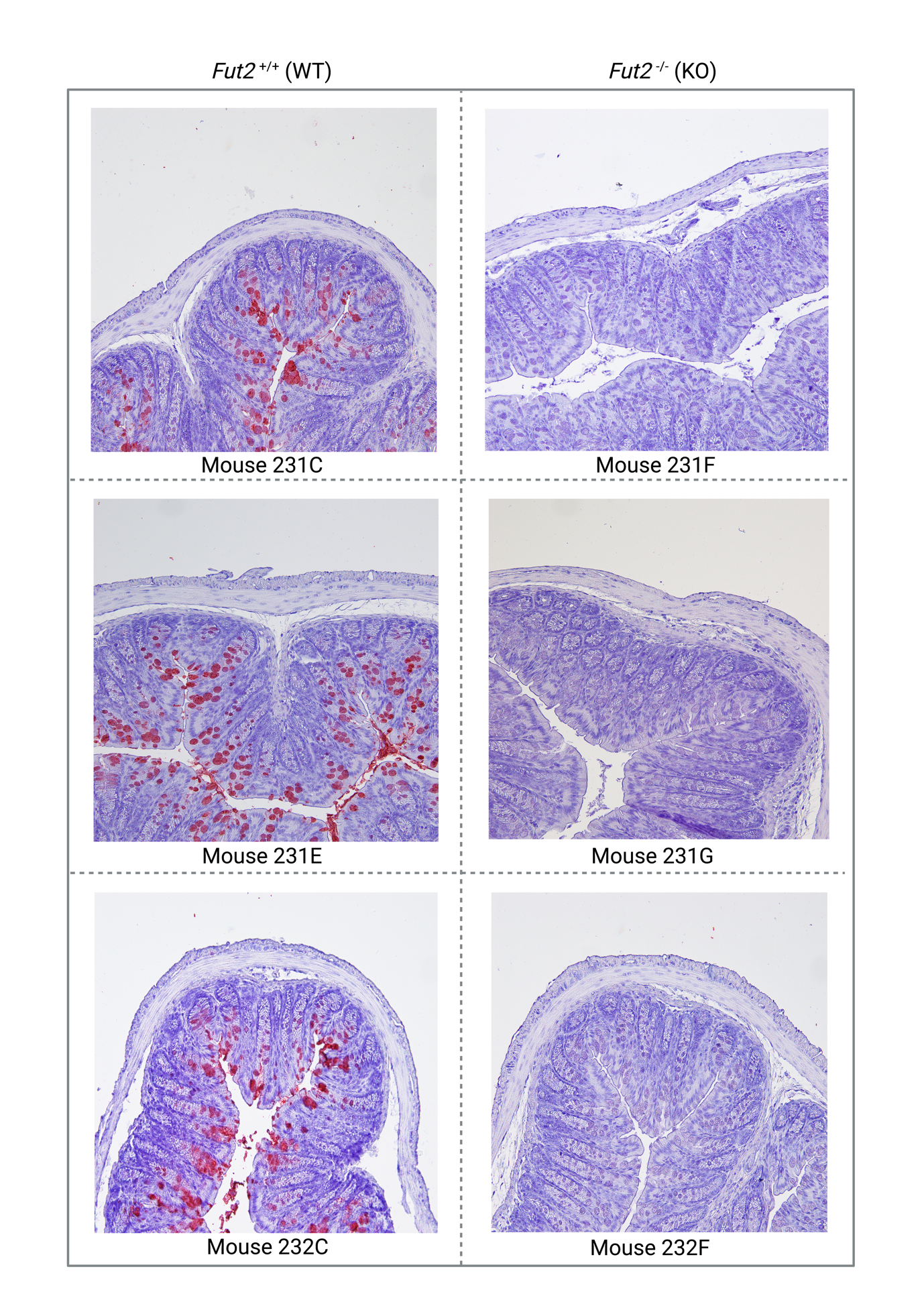


**Fig. S1.** Representative images of large intestine tissue from *Fut2*^WT^ and *Fut2*^KO^ mice stained with HRP-conjugated UEA-1 lectin. Confirming loss of α(1,2)-fucosylated glycan production in KO mice.


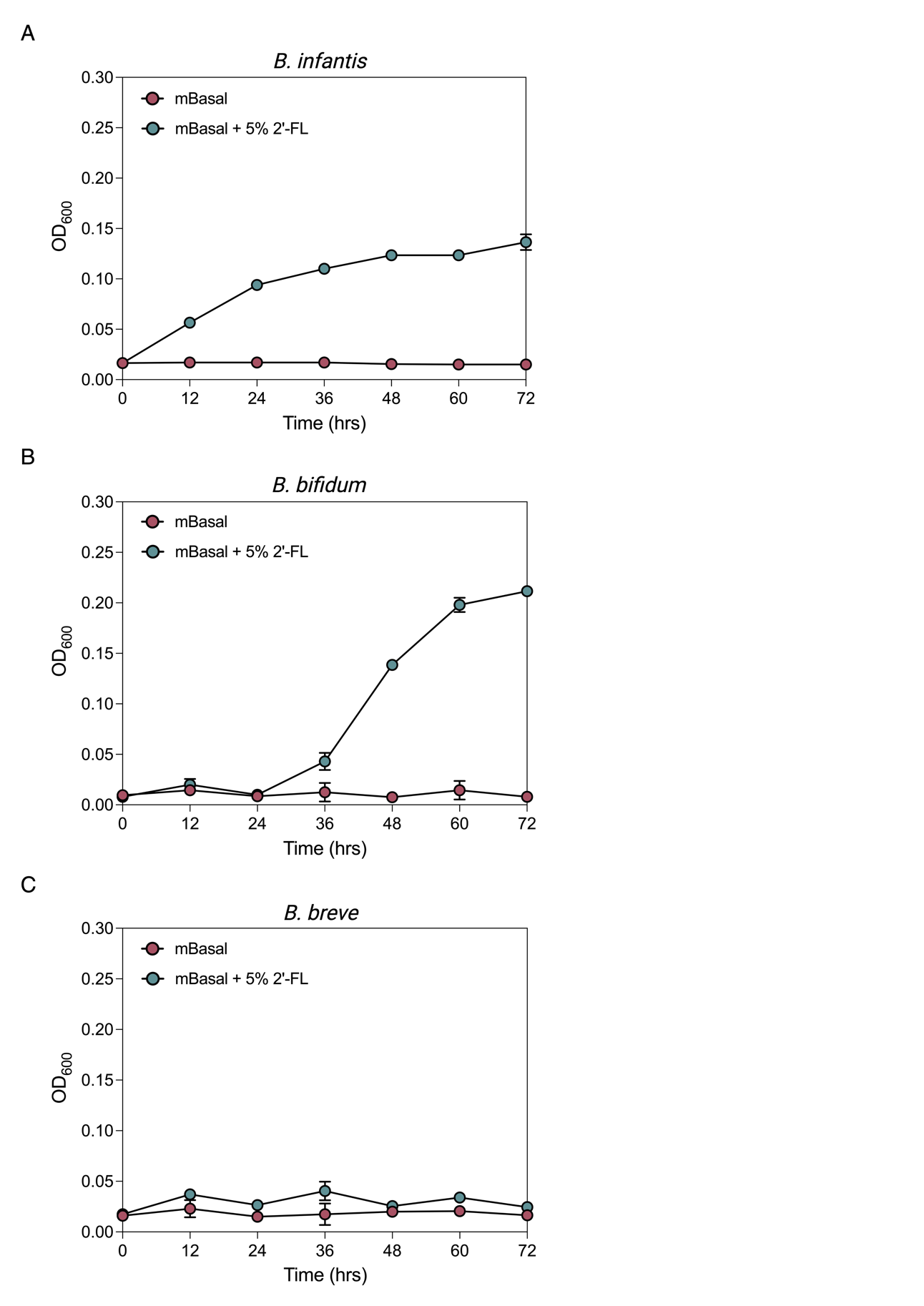


**Fig. S2** Anaerobic *in vitro* growth of *Bifidobacterium* species either with or without 2ˈ-fucosyllactose (2ˈ-FL) supplementation. Showing immediate enhanced growth of *B. infantis* with supplementation, reflecting genetic ability to internalise and metabolise 2ˈ-FL. *B. bifidum* shows longer duration until enhanced growth. *B. breve* shows no enhanced growth with 2ˈ-FL.


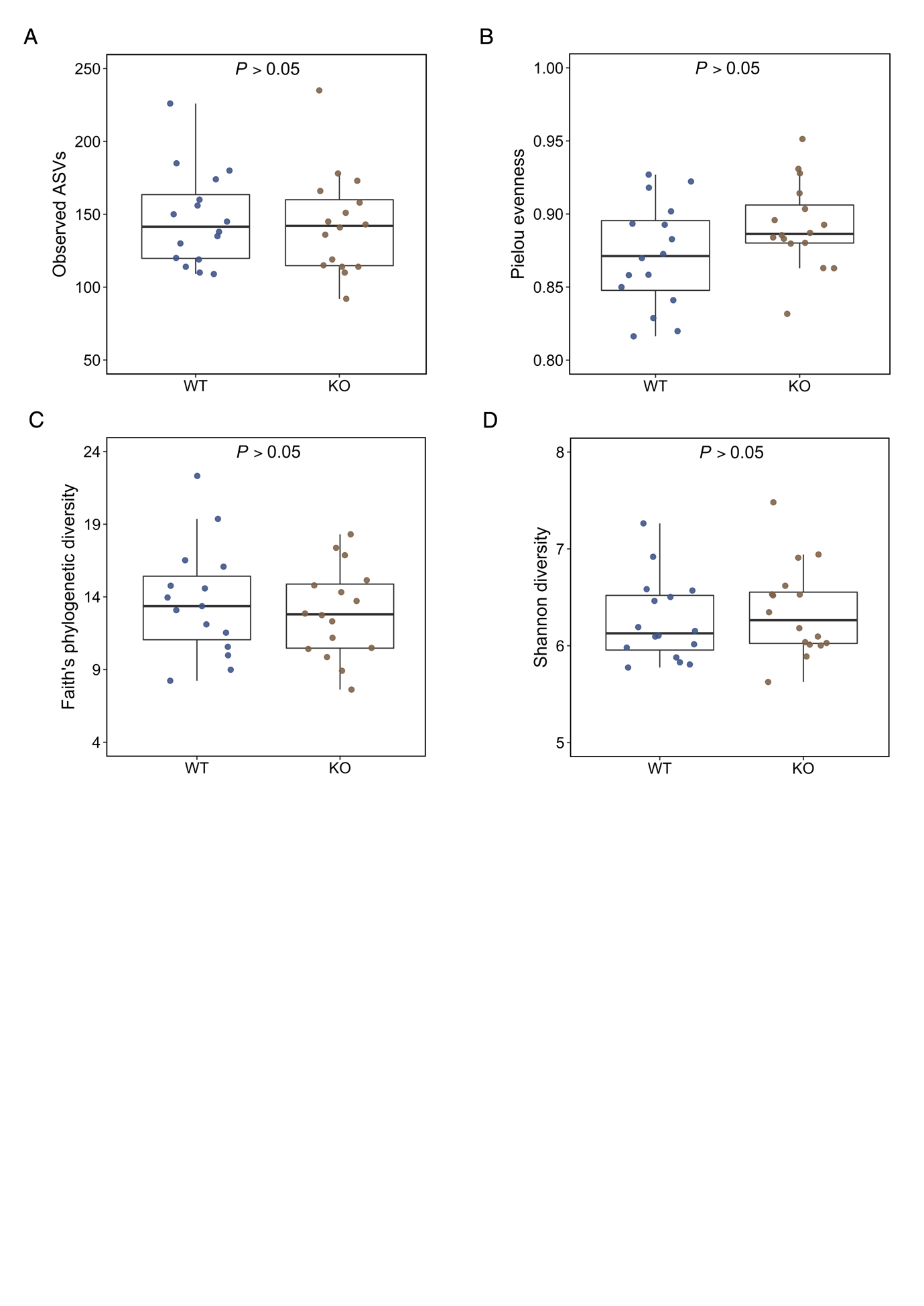


**Fig. S3** Faecal microbiota α-diversity in male *Fut2*^WT^ and *Fut2*^KO^ mice.


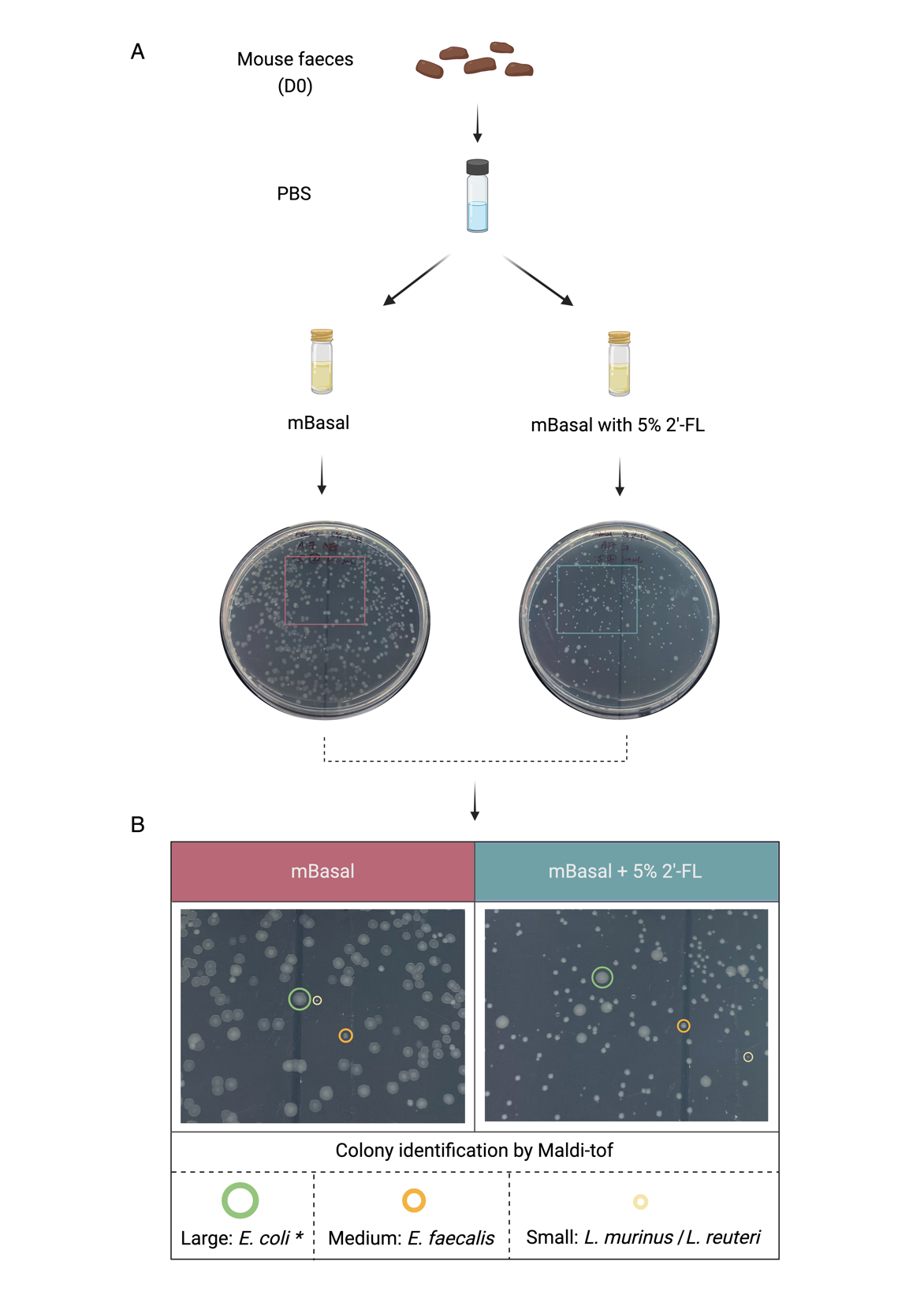


**Fig. S4** Anaerobic *in vitro* growth of faecal bacteria from *Fut2*^WT^ mice either with or without 2ˈ-fucosyllactose (2ˈ-FL) supplementation. Colonies characterised by size as small medium and large, with representative colony identification confirmed by matrix-assisted laser desorption/ionization time-of-flight mass spectrometry (MALDI-TOF).


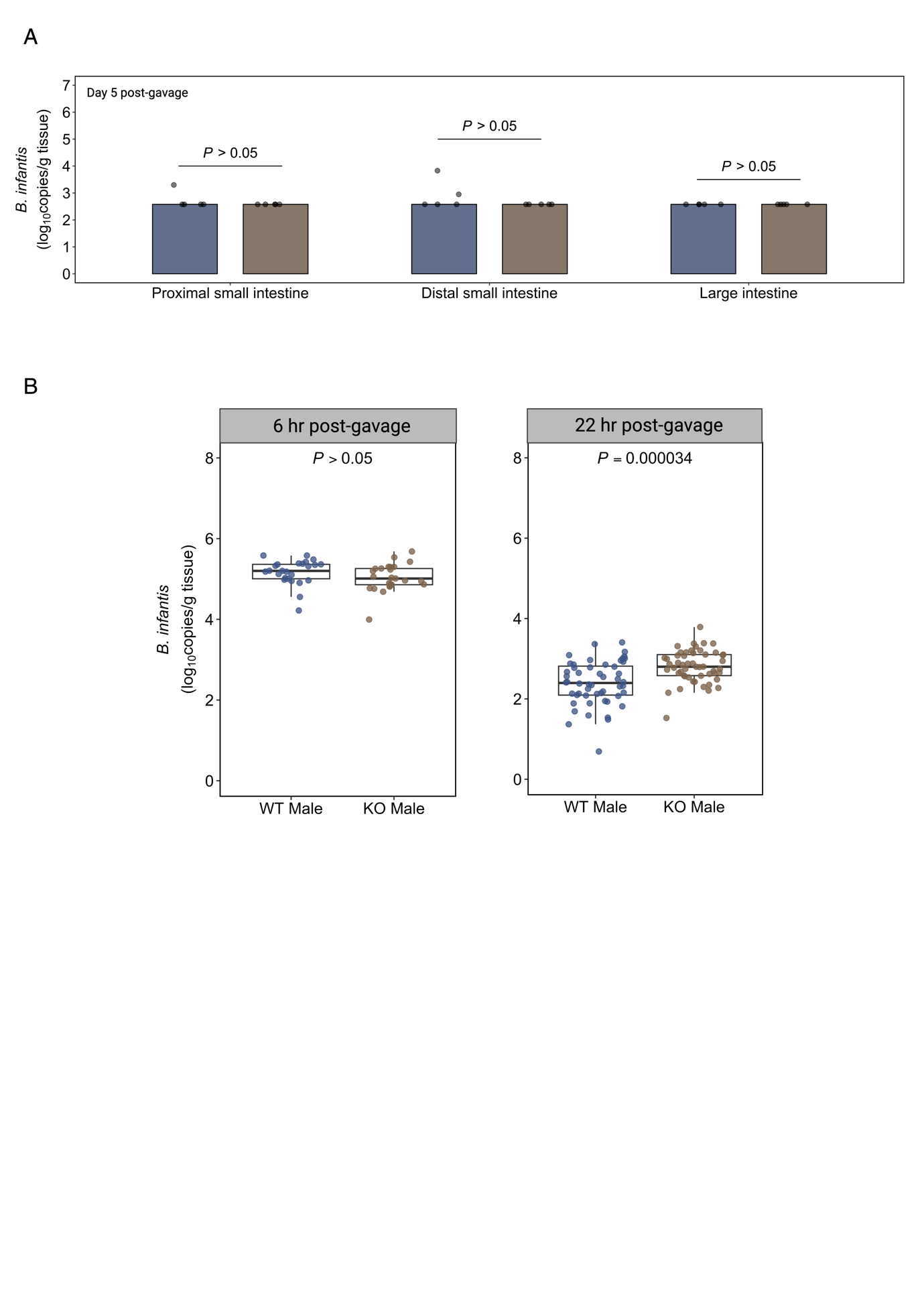


**Fig. S5** Abundance of *Bifidobacterium infantis* of *Fut2*^WT^ and *Fut2*^KO^ mice following *B. infantis* gavage. A) Intestinal mucosal tissue 5 days post *B. infantis* gavage. B) Faecal *B. infantis* abundance during gavage period. Showing both *Fut2*^WT^ and *Fut2*^KO^ have similarly high levels of *B. infantis* 6 hours after gavage, however *Fut2*^KO^ mice have significantly higher *B. infantis* abundance at 22 hours after gavage.

**References**

1. Choo JM, Leong LE, Rogers GB; Sample storage conditions significantly influence faecal microbiome profiles. *Sci Rep* 2015;**5**:16350. doi: 10.1038/srep16350.

2. Bolyen E, Rideout JR, Dillon MR, Bokulich NA, Abnet CC, Al-Ghalith GA, et al.; Reproducible, interactive, scalable and extensible microbiome data science using QIIME 2. *Nat Biotechnol* 2019;**37**(8):852-857. doi: 10.1038/s41587-019-0209-9.

3. Nadkarni MA, Martin FE, Jacques NA, Hunter N; Determination of bacterial load by real-time PCR using a broad-range (universal) probe and primers set. *Microbiology* 2002;**148**(1):257-266. doi: doi:10.1099/00221287-148-1-257.

4. Haarman M, Knol J; Quantitative real-time PCR assays to identify and quantify fecal Bifidobacterium species in infants receiving a prebiotic infant formula. *Appl Environ Microbiol* 2005;**71**(5):2318-24. doi: 10.1128/AEM.71.5.2318-2324.2005.

5. Junick J, Blaut M; Quantification of human fecal bifidobacterium species by use of quantitative real-time PCR analysis targeting the groEL gene. *Appl Environ Microbiol* 2012;**78**(8):2613-22. doi: 10.1128/AEM.07749-11.
